# The anti-cancer effect of betulinic acid in u937 human leukemia cells is mediated through ROS-dependent cell cycle arrest and apoptosis

**DOI:** 10.1080/19768354.2021.1915380

**Published:** 2021-04-23

**Authors:** Cheol Park, Jin-Woo Jeong, Min Ho Han, Hyesook Lee, Gi-Young Kim, Soojung Jin, Jung-Ha Park, Hyun Ju Kwon, Byung Woo Kim, Yung Hyun Choi

**Affiliations:** a College of Liberal Studies, Dong-Eui University, Busan, Republic of Korea; b Nakdonggang National Institute of Biological Resources, Sangju, Republic of Korea; c National Marine Biodiversity Institute of Korea, Seocheon, Republic of Korea; d Anti-Aging Research Center, Dong-eui University, Busan, Republic of Korea; e Department of Biochemistry, Dong-eui University College of Korean Medicine, Busan, Republic of Korea; f Department of Marine Life Sciences, Jeju National University, Jeju, Republic of Korea; g Core-Facility Center for Tissue Regeneration, Dong Eui University, Busan, Republic of Korea; h Biopharmaceutical Engineering Major, Dong-eui University, Busan, Republic of Korea

**Keywords:** Betulinic acid, leukemia cells, G2/M arrest, apoptosis, ROS

## Abstract

Although previous studies have shown anti-cancer activity of betulinic acid (BA), a pentacyclic triterpenoid, against various cancer lines, the underlying molecular mechanisms are not well elucidated. In this study, we evaluated the mechanisms involved in the anti-cancer efficacy of BA in U937 human myeloid leukemia cells. BA exerted a significant cytotoxic effect on U937 cells through blocking cell cycle arrest at the G2/M phase and inducing apoptosis, and that the intracellular reactive oxygen species (ROS) levels increased after treatment with BA. The down-regulation of cyclin A and cyclin B1, and up-regulation of cyclin-dependent kinase inhibitor p21WAF1/CIP1 revealed the G2/M phase arrest mechanism of BA. In addition, BA induced the cytosolic release of cytochrome *c* by reducing the mitochondrial membrane potential with an increasing Bax/Bcl-2 expression ratio. BA also increased the activity of caspase-9 and -3, and subsequent degradation of the poly (ADP-ribose) polymerase. However, quenching of ROS by *N*-acetyl-cysteine, an ROS scavenger, markedly abolished BA-induced G2/M arrest and apoptosis, indicating that the generation of ROS plays a key role in inhibiting the proliferation of U937 cells by BA treatment. Taken together, our results provide a mechanistic rationale that BA exhibits anti-cancer properties in U937 leukemia cells through ROS-dependent induction of cell cycle arrest at G2/M phase and apoptosis.

## Introduction

Leukemia, with the abnormal proliferative properties of white blood cells, is one of the most common blood cancers and continues to increase worldwide (Pelland-Marcotte et al. 2019; Moore et al. 2020). Currently, chemotherapy, radiation therapy, and hematopoietic stem cell transplantation are being applied as general clinical therapies for the treatment of leukemia patients. However, the poor prognosis, drug resistance and serious side effects are hurdles to effective treatment and lead to frequent recurrences (Shimada 2019; Bárcenas-López et al. 2021). Therefore, there is an urgent need to develop a new therapeutic agent with low side effects and high efficiency for the treatment of leukemia.

Betulinic acid (BA) is a naturally pentacyclic triterpenoid that is widely distributed throughout the plant kingdom, especially abundant in birch bark (*Betula* sp.) (Rastogi et al. 2015; Ríos and Máñez 2018). Accumulated evidence demonstrates that BA possesses various biological activities, including antioxidant, anti-inflammatory, hepatoprotective, and anti-tumor effects (Saneja et al. 2018; An et al., 2020; Li et al. 2021; Kong et al. 2021). Among them, the anti-tumor potential has recently received great attention, showing that the anti-cancer mechanisms of BA are complex and depends on the type of cancer cells, without causing toxicity toward normal cells (Mullauer et al. 2010; Gheorgheosu et al. 2014; Kumar et al. 2018; Zuco et al. 2020; Kutkowska et al. 2021). For example, BA inhibited cell viability and induced apoptosis in the G0/G1 phase of cell cycle progression in human breast cancer, oral squamous cell carcinoma, cervical cancer, lung cancer, leukemia cells, etc. (Xu et al. 2017; Goswami et al. 2018; Shen et al. 2019). On the other hand, BA triggered apoptosis by arresting the cell cycle at the S or G2/M stage in certain myeloma, gastric and lung cancer cell lines (Rzeski et al. 2006; Zhan et al. 2018). Interestingly, Shen et al. (2019) recently reported that the suppression of the nuclear factor-kappa B pathway increased downstream oxidant effectors, thereby promoting the generation of reactive oxygen species (ROS) in BA-stimulated multiple myeloma cells. Although BA is known to have antioxidant activity that blocks the accumulation of ROS due to oxidative stress in normal cells (Cheng et al. 2019; Cheng et al. 2019; Kong et al. 2021), their results well support the previous studies that BA promotes ROS production in cancer cells (Wang et al. 2017, 2019; Xu et al. 2017; Goswami et al. 2018; Shen et al. 2019). However, the underlying anti-cancer mechanism of BA and associated molecular targets and the role of ROS are poorly identified in multiple cancer types. Therefore, in this study, we investigated the mechanisms involved in the effect of BA on the growth inhibition of U937 human myeloid leukemia cells.

## Materials and methods

### Cell culture and BA treatment

U937, Jurkat (human T cell acute lymphocytic leukemia), C2C12 (mouse myoblast cell line), and V79-4 (Chinese hamster lung fibroblast cell line) cells were purchased from the American Type Culture Collection (Manassas, VA, USA). The cells were cultured in Dulbecco’s modified Eagles medium (U937, C2C12, and V79-4 cells) or RPMI-1640 medium (Jurkat cells) supplemented with 10% fetal bovine serum and antibiotics (WelGENE Inc., Gyeongsan, Republic of Korea) in a humidified atmosphere of 5% CO_2_ and 95% air at 37°C. BA (Sigma-Aldrich Chemical Co., St. Louis, MO, USA) was dissolved in dimethyl sulfoxide (DMSO) to prepare a stock solution. The stock solution was diluted with culture medium before use in the experiments.

### Cell viability assay

Cell viability was measured by the 3-(4,5-dimethylthiazol-2-yl)-2,5-diphenyltetrazolium bromide (MTT) reduction assay (Choi 2020). Briefly, the cells were treated with BA for 48 h in the absence or presence of *N*-acetyl-cysteine (NAC, Sigma-Aldrich Chemical Co.). Thereafter, MTT solution (Sigma-Aldrich Chemical Co.) was added and incubated for 3 h. After removing the supernatant, DMSO was added to dissolve the formed formazan crystals. The absorbance was quantified at a wavelength of 540 nm using an enzyme-linked immunosorbent assay (ELISA) plate reader (Molecular Device Co., Sunnyvale, CA, USA).

### Measurement of ROS generation

The production of ROS was detected by 2′,7′-dichlorohydrofluoresce in diacetate (DCF-DA) staining. After treatment with BA for the indicated times with or without NAC, the cells were stained with DCF-DA (Sigma-Aldrich Chemical Co.) at 37°C℃ for 30 min. Then, the cells were rinsed with phosphate-buffered saline (PBS) and ROS levels were quantified by flow cytometry (BD Biosciences, San Jose, CA, USA) as previously described (Choi 2020).

### Cell cycle analysis

After treatment for 48 h with BA in the absence or presence of NAC, the cells were stained with propidium iodide (PI, Sigma-Aldrich Chemical Co.) following fixation with ice-cold 70% ethanol as previously described (Choi 2020). After incubation for 30 min, the cell cycle distribution was analyzed using flow cytometry.

### Protein extraction and Western blot analysis

The cells were collected and lysed as previously described (Choi 2020). Protein samples were separated by sodium dodecyl sulfate-polyacrylamide gel electrophoresis, transferred to polyvinylidene fluoride membranes (Millipore, Bedford, MA, USA), and then incubated with the primary antibodies at 4°C overnight. Thereafter, the membranes were incubated with the secondary antibodies at room temperature (RT) for 2 h. The immune-reactive bands were visualized using an enhanced chemiluminescent (Thermo Fisher Scientific, Waltham, MA, USA). Antibodies used in this study were purchased from Santa Cruz Biotechnology Inc. (Santa Cruz, CA, USA), and Cell Signaling Technology (Beverly, MA, USA).

### Detection of apoptotic cells by flow cytometry

The cells were collected and resuspended in the suspension buffer provided in the kit (Annexin V-fluorescein isothiocyanate (FITC) Apoptosis Detection Kit, BD Biosciences). After that, the cells were stained with Annexin V/PI and then analyzed by flow cytometry (Choi 2020).

### DNA fragmentation assay

The cells were washed with PBS, resuspended in lysis buffer and incubated at RT for 20 min as described by Choi (2020). After that, DNA was isolated from the cells and electrophoresed on a 1.5% agarose gel, and the gel was stained with ethidium bromide (EtBr, Sigma-Aldrich Chemical Co.) and observed with UV transillumination.

### Detection of apoptotic morphological changes

After the termination of treatment, the cells were washed with PBS, fixed using 4% paraformaldehyde for 10 min at RT, and then stained with 4′,6-diamidino-2-phenylindole (DAPI, Sigma-Aldrich Chemical Co.) for 15 min in the dark. The pattern of changes in nuclear morphology was observed using a fluorescence microscope (Carl Zeiss, Oberkochen, Germany).

### Caspase activity assay

Caspase activities were measured using colorimetric assay kits (R&D Systems, Minneapolis, MN, USA). In brief, the equal amounts of proteins were incubated with the reaction buffer with the appropriate caspase fluorogenic substrates for 2 h at 37°C according to the manufacturer’s instructions.

### Measurement of mitochondrial membrane potential (MMP, ΔΨm)

The levels of MMP were assessed by 5,5′6,6′-tetrachloro-1,1′,3,3′-tetraethyl-imidacarbocyanine iodide (JC-1) staining. The collected cells were stained with JC-1 solution (Sigma-Aldrich Chemical Co.) for 20 min at 37°C, and then measured using a flow cytometer (Choi 2020).

### Statistical analysis

All experiments were carried out in triplicate and results were expressed as the means ± standard deviation (SD) of the obtained results. Graph Pad Prism 5.03 software (GraphPad Software, Inc., La Jolla, CA, USA) was used for statistical analysis. A value *p *< 0.05 was considered statistically significant.

## Results

### BA reduces the viability of U937 cells in a ROS-dependent manner

**Figure 1.**
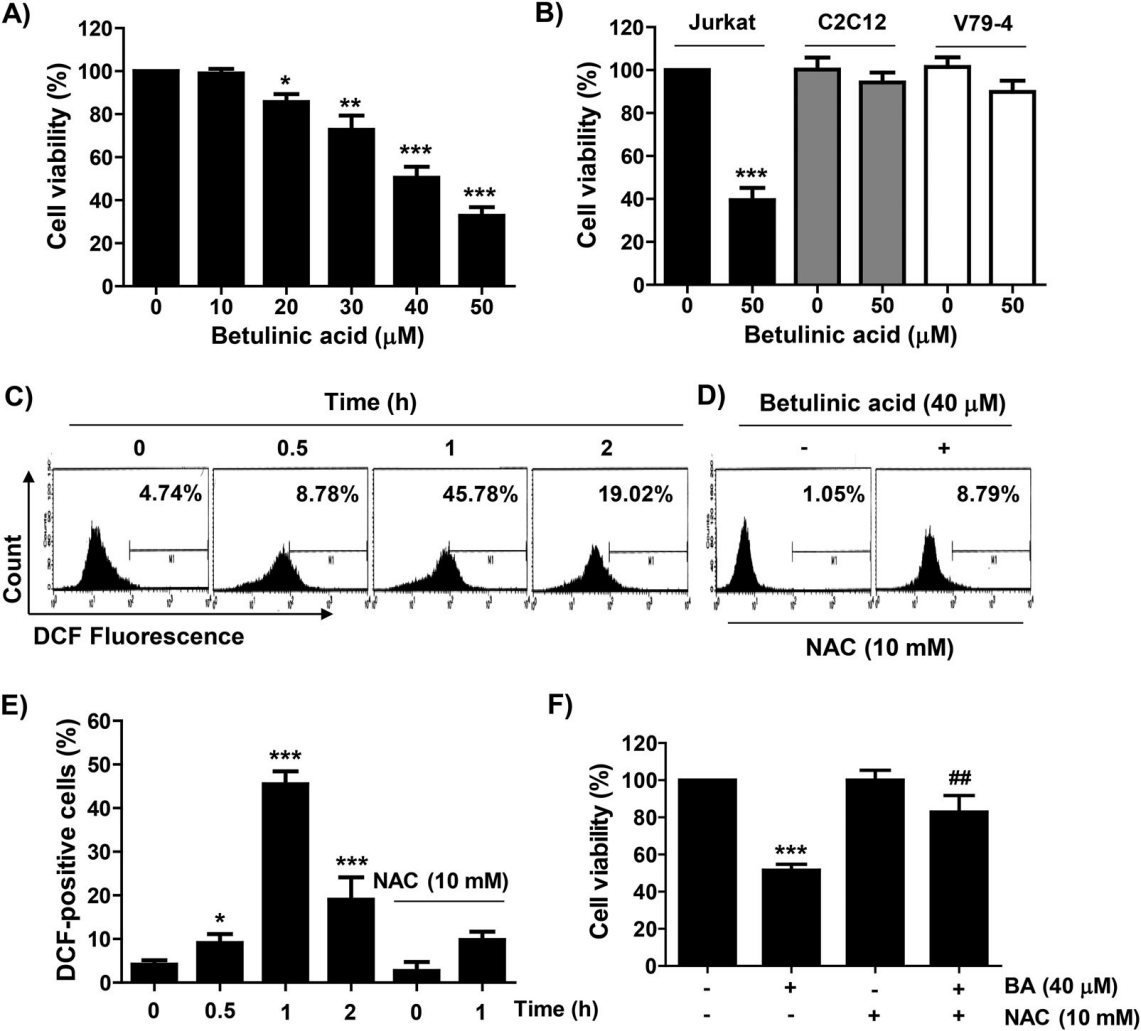
Effect of BA on the cell viability of U937 cells. (A and B) U937, Jurkat, C2C12, and V79-4 cells were treated with the indicated concentrations of BA for 48 h. (C–F) U937 cells were pre-treated with or without NAC for 1 h, and then treated with BA for the indicated times (C–E) or 48 h (F). (A, B and F) Cell viability was measured by the MTT assay. (C–E) After staining with DCF-DA, the levels of ROS were measured. (C and D) Representative profiles of the results of flow cytometry. (B, E and F) Results were expressed as the mean ± SD (*p < 0.05, **p < 0.01, and ***p < 0.001 *vs* untreated cells. ^##^*p* < 0.01 *vs* BA-treated cells).

BA significantly reduced the viability of U937 cells in a concentration-dependent manner when compared with the untreated cells (Figure 1(A)). A similar decrease in proliferation was observed in Jurkat cells. However, in normal cell lines including C2C12 myoblasts and V79-4 lung fibroblasts, only a slight decrease in viability was observed (Figure 1(B)). In addition, in the BA-treated U937 cells, ROS production increased from the initial time point of BA treatment and showed a peak after 1 h. However, BA-induced ROS production was markedly blocked by the pre-treatment of NAC, an ROS scavenger, and the reduced viability of U937 cells by BA was significantly offset in the presence of NAC (Figures 1(C–F)).

### BA induces cell cycle arrest at the G2/M phase in a ROS-dependent manner in U937 cells

**Figure 2.**
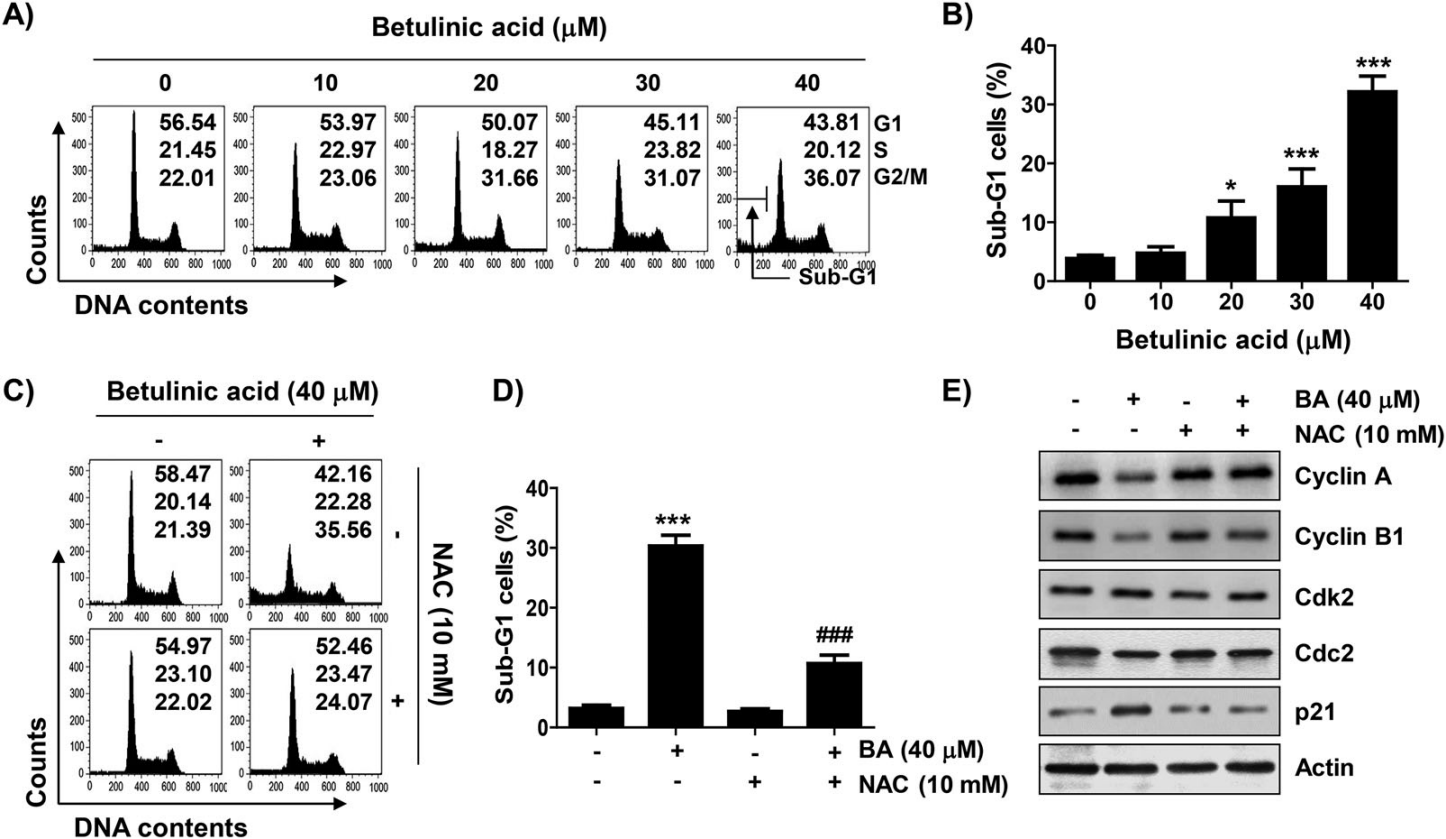
Induction of G2/M arrest by BA in U937 cells. Cells were treated with different concentrations of BA for 48 h (A and B) or pre-treated with or without NAC for 1 h, and then treated with BA for 48 h (C–E). (A and C) Representative flow cytometric histograms for each cell cycle phase and their relative ratios. (B and D) The percentages of the sub-G1 phase cells. (B and D) Results were expressed as the mean ± SD (*p < 0.05, and ***p < 0.001 *vs* untreated cells, ^###^*p* < 0.001 *vs* BA-treated cells). (E) Changes in the expression of cell cycle regulatory proteins were detected. Actin was used as a loading control.

As shown in Figure 2(A), the proportion of cells in the G2/M phase in BA-treated cells gradually increased in a concentration-dependent manner, while those of cells in the G1 and S phases were relatively decreased. Meanwhile, the frequency of cells belonging to the sub-G1 phase, an indicator of apoptosis, significantly increased in a concentration-dependent manner of BA (Figure 2(B)). Subsequently, immunoblotting results showed that the addition of BA did not change the expression of cyclin-dependent kinase 2 (Cdk2) and cell division control protein 2 (Ccd2), but the levels of cyclin A and cyclinB1 were considerably reduced. On the other hand, Cdk inhibitor p21WAF1/CIP1 was markedly upregulated. However, when the production of ROS was blocked by NAC, BA-induced cell cycle arrest was completely reversed. And, the expression of the cell cycle regulators, which was changed by BA treatment, was maintained at the control level in the presence of NAC (Figures 2(C–E)).

### Induction of apoptosis by BA is ROS dependent in U937 cells

**Figure 3.**
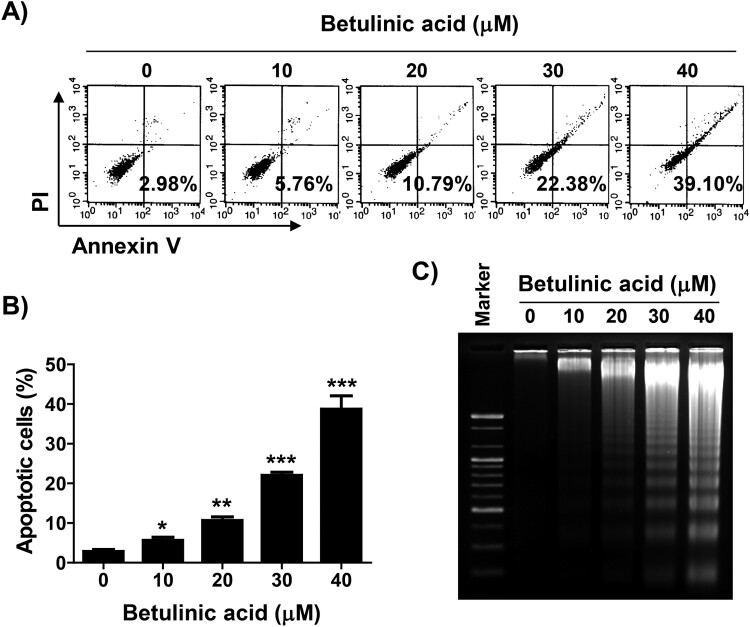
Induction of apoptosis by BA in U937 cells. Cells were treated with the indicated concentrations of BA for 48 h. (A and B) The percentage of apoptotic cells, defined as annexin V-positive cells, was detected. (A) Representative flow cytometric histograms. (B) The percentages of apoptotic cells. Results were expressed as the mean ± SD (*p < 0.05, **p < 0.01, and ***p < 0.001 *vs* untreated cells). (C) The extracted genomic DNA was separated in an agarose gel, stained with EtBr and then visualized.

**Figure 4.**
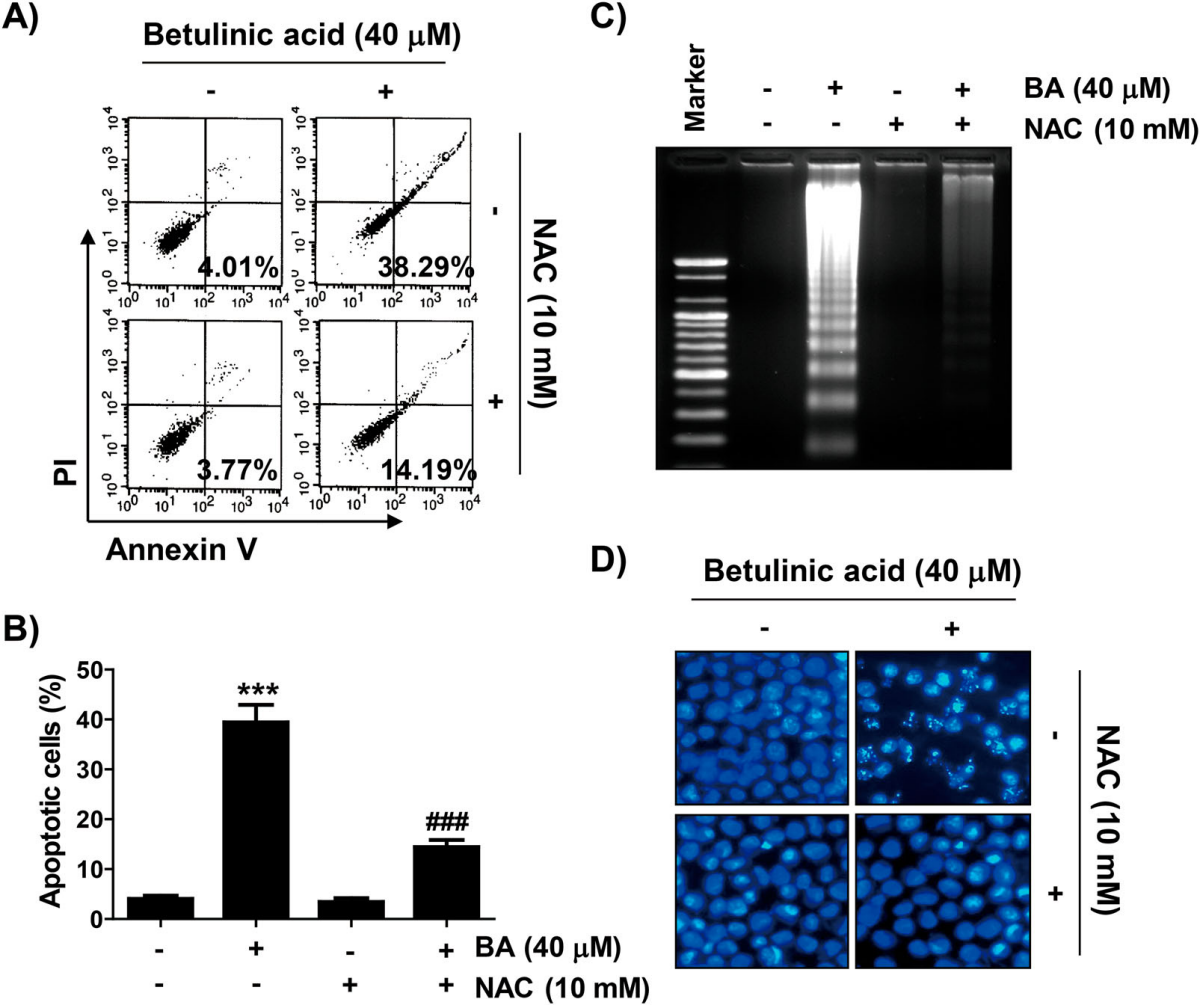
Role of ROS in the induction of apoptosis by BA in U937 cells. Cells were pre-treated with or without NAC for 1 h, and then treated with BA for 48 h. (A and B) Cells were stained with Annexin V and PI. (A) Representative flow cytometric histograms. (B) The percentages of apoptotic cells. Results were expressed as the mean ± SD (***p < 0.001 *vs* untreated cells, ^###^*p* < 0.001 *vs* BA-treated cells). (C) DNA fragmentation assay was performed. (D) DAPI staining was performed to analyze the morphological characteristics of the nuclei.

Annexin V/PI double staining and agarose gel electrophoresis were performed to investigate whether the inhibition of U937 proliferation by BA was associated with the induction of apoptosis. As shown in Figures 3(A–C), BA treatment increased the frequency of Annex in V-positive cells and fragmented DNA bands compared to the control in a concentration-dependent manner. Next, we confirmed whether BA-induced apoptosis was related to ROS production, and found that BA-induced apoptosis was markedly attenuated by NAC by observing the results of flow cytometry analysis and agarose gel electrophoresis as well as changes in nuclear morphology by DAPI staining (Figure 4).

### BA increases the activity of caspase-9 and -3, but not caspase-8, in U937 cells

**Figure 5.**
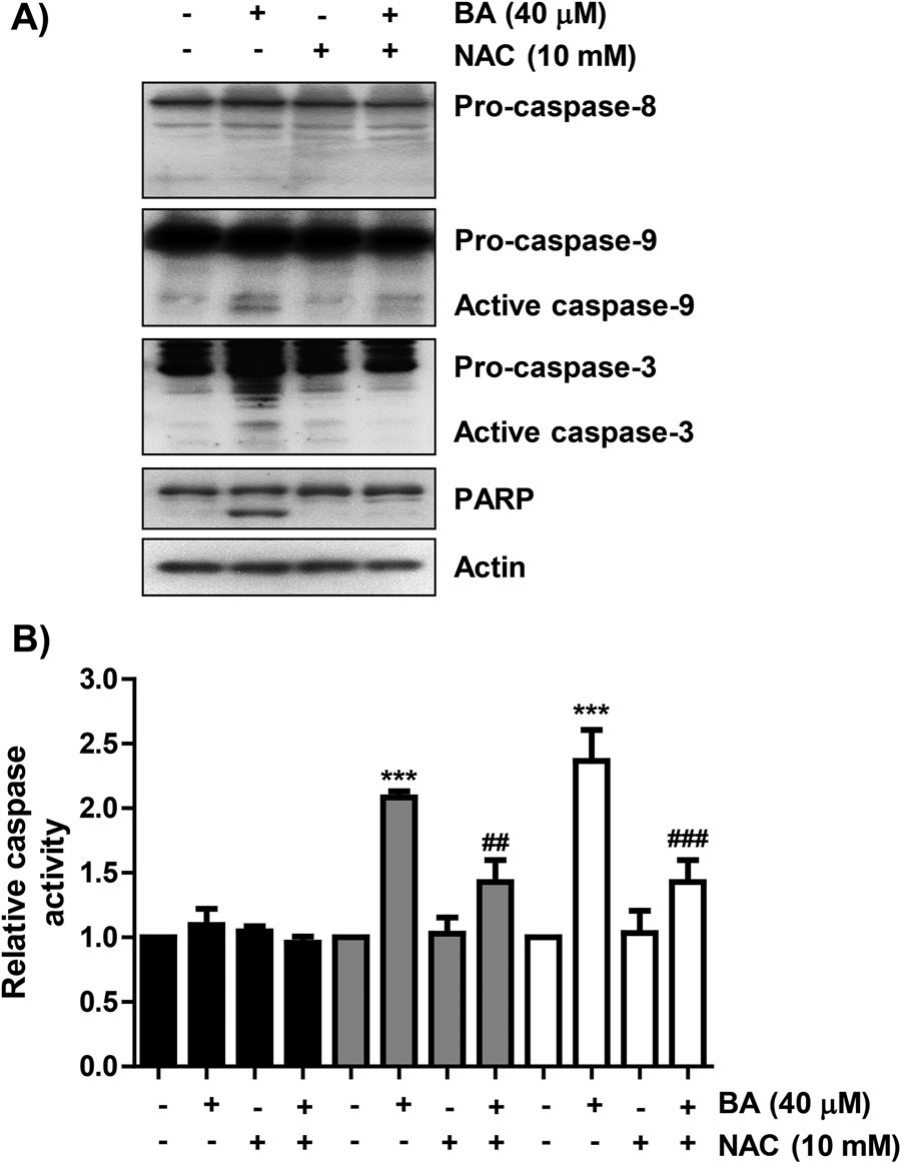
Activation of caspase-9 and -3 in BA-treated U937 cells. Cells were either treated with BA for 48 h, or treated with NAC for 1 h before BA treatment. (A) Changes in the expression of caspases and PARP were detected. (B) The activities of caspases were measured by caspase colorimetric assay kits. Results were expressed as the mean ± SD (***p < 0.001 *vs* untreated cells, ^##^*p* < 0.01 and ^###^*p* < 0.001 *vs* BA-treated cells).

We next investigated whether BA-induced apoptosis was associated with the activation of caspase cascade, and found that the expression of the active forms of caspase-9 and -3, and their activity were significantly increased in BA-treated cells (Figure 5). At the same time, degradation of poly (ADP-ribose) polymerase (PARP) was induced in BA-treated cells, but caspase-8 was not activated. However, in the presence of NAC, the activation of caspase-9 and -3 were attenuated, and the expression of PARP showed a similar tendency to that of the control group.

### BA triggers ROS-dependent mitochondrial impairment in U937 cells

**Figure 6.**
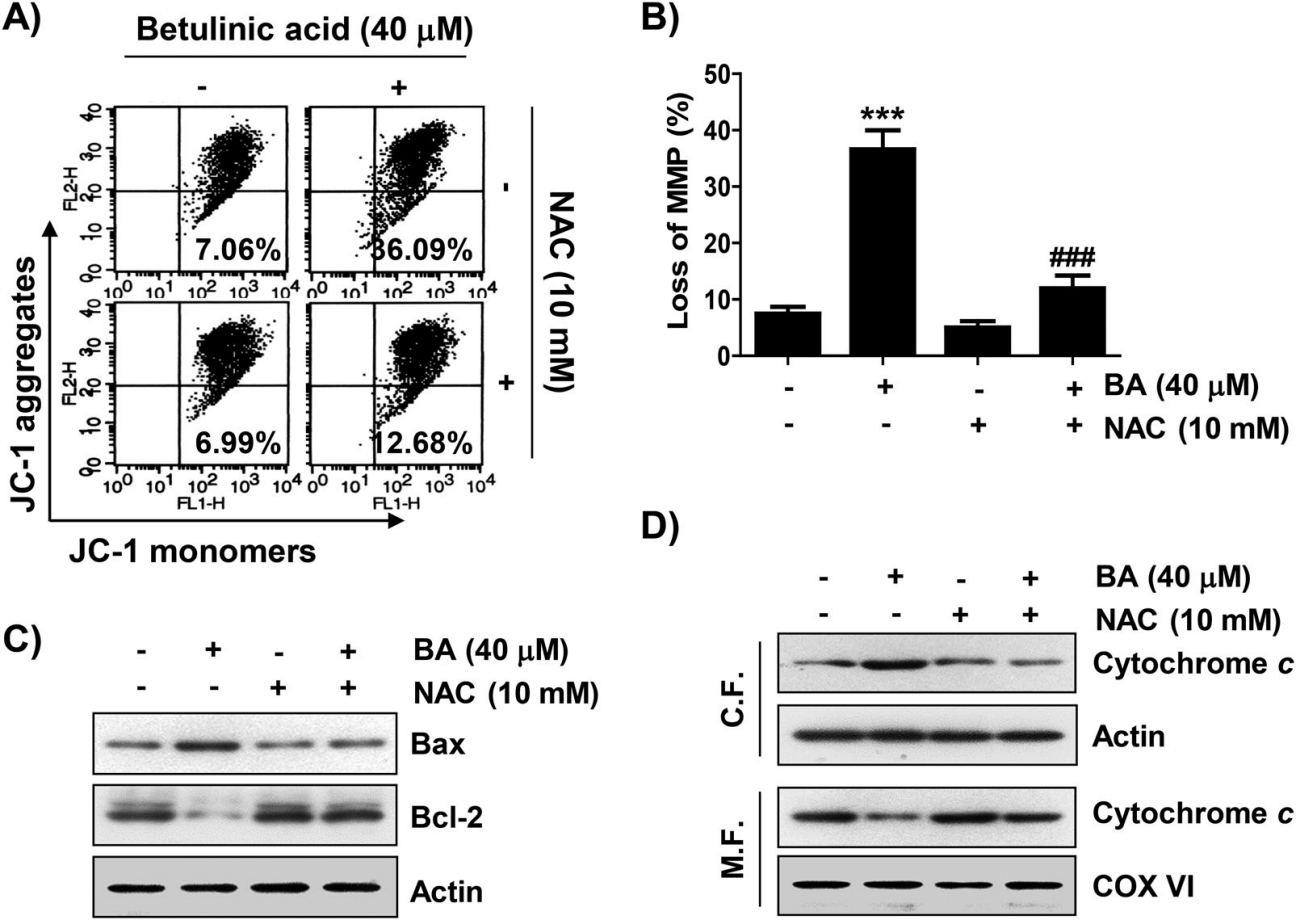
ROS-dependent impairment of mitochondrial function in BA-treated U937 cells. Cells were pre-treated with or without NAC for 1 h, and then treated with BA for 48 h. (A and B) Cells were stained with JC-1. (A) Representative flow cytometric histograms were presented. (B) The percentages of depletion of MMP. Results were expressed as the mean ± SD (***p < 0.001 *vs* untreated cells, ^###^*p* < 0.001 *vs* BA-treated cells). (C) Changes in the expression of Bax and Bcl-2 were detected. (D) Cytochrome *c* expression was analyzed by Western blotting. Protein loading was confirmed by the analysis of actin and cytochrome oxidase subunit VI (COX VI) expression, respectively. CF, cytosolic fraction; MF, mitochondrial fraction.

We further evaluated whether apoptosis by BA was involved in mitochondrial dysfunction. As shown in Figures 6(A,B), the level of JC-1 monomer, an indicator of MMP depletion, was significantly increased in cells after BA treatment, but pre-treatment with NAC suppressed BA-mediated MMP depletion. BA markedly also upregulated and downregulated the expression of pro-apoptotic Bax and anti-apoptotic Bcl-2, respectively, and NAC rescued their levels (Figure 6(C)). Moreover, BA enhanced the release of cytochrome *c* from the mitochondria to the cytoplasm, which was also completely inhibited by blocking ROS production (Figure 6(D)).

## Discussion

In this study, we investigated the anti-proliferative effect of BA in U937 leukemia cells, and found that BA reduced U937 cell viability through the regulation of the expression of determinants that control cell cycle progression and apoptosis. In particular, BA-induced cell cycle arrest and apoptosis were accompanied by the loss of mitochondrial integrity initiated by excessive ROS production, suggesting a redox-dependent mechanism.

The level of ROS in the cells is a critical factor that regulates cell proliferation and cell death. Excessive accumulation of ROS can lead to cell cycle arrest and apoptosis through the regulation of various pathways (Moloney and Cotter 2018; Rodrigues and Ferraz 2020). After referencing a lot of literature, we found that BA can induce ROS formation in cancer cells (Wang et al. 2017, 2019; Xu et al. 2017; Goswami et al. 2018; Shen et al. 2019), but may reduce ROS production in normal cells (Cheng et al. 2019; Cheng et al. 2019; Kong et al. 2021), which means that BA has potent antioxidant activity in normal cells. Therefore, we measured the ROS content after BA treatment and found that BA was able to effectively increase ROS production at the initial time point of BA treatment, which was directly involved in BA-induced G2/M phase arrest and apoptosis. Cell cycle progression is a series of events that control the growth and proliferation of new cells (Yun et al. 2013; Çoban et al. 2020). In the progression of the cell cycle, the G1/S and G2/M stages are important checkpoints and are thoroughly regulated by various cell cycle regulators including cyclins, Cdks and Cdk inhibitors. For cells to proliferate, they must enter the G1/S phase for DNA synthesis and centrosome replication, followed by the G2/M phase for preparation for mitosis, and division. In particular, the activity of the Cdk2/cyclin A complex is required for precursor initiation during the G2/M transition, and the Cdc2/cyclin B complex actively participates in and completes the M phase (Satyanarayana and Kaldis 2009; Çoban et al. 2020). According to the results of this study, the expression of p21WAF1/CIP1, a representative Cdk inhibitor, markedly increased by BA treatment, whereas cyclin A and cyclin B1 reduced without changing the expression of Cdk2 and Cdc2. Furthermore, we used the ROS scavenger, NAC, to confirm whether ROS affected BA-induced cytotoxicity, and found that NAC was able to block the reduction of viability and G2/M phase arrest in BA-treated cells by reducing ROS production. Expression of cell cycle regulators changed by BA treatment was also maintained at the control level by blocking ROS production, suggesting that BA exerts anti-proliferative and cell cycle arrest effects in U937 cells, at least through ROS generation.

As is well known, apoptosis-inducing pathways can be divided into the death receptor (DR)-mediated extrinsic pathway and the mitochondria-related intrinsic pathway. The extrinsic pathway is triggered by the binding of death ligands to DRs in the outer cell membrane, followed by caspase-8 is activated. The intrinsic pathway is associated with changes in the expression of Bcl-2 family proteins and the release of cytochrome *c* into the cytoplasm following impaired mitochondrial function, and caspase-9 is activated when this pathway is initiated by stimulation of apoptosis (Kiraz et al. 2016; D’Arcy 2019). Activated caspase-8 and -9 induce caspase-3 activation, and subsequently led to cleavage of various substrates, including PARP, thereby accelerating apoptosis (Edlich 2018; D’Arcy 2019). Our results showed that BA induced the depletion of MMP, cytosolic release of cytochrome *c*, and an increase in the Bax/Bcl-2 expression ratio. BA also enhanced the activity of caspase-9 and -3, but not caspase-8, and induced the degradation of PARP, indicating that BA promoted apoptosis in U937 cells through the activation of the intrinsic pathway. Meanwhile, disturbance of mitochondrial membrane permeability due to mitochondrial dysfunction caused by excessive ROS production trigger apoptosis through the activation of the intrinsic pathway (Moloney and Cotter 2018; Rodrigues and Ferraz 2020). In this study, BA-induced loss of MMP was significantly suppressed when ROS production was blocked. In addition, quenching of ROS fundamentally protected changes in the expression of mitochondria-mediated apoptosis regulators by BA, which clearly indicates that BA induced apoptosis in U937 cells by promoting ROS generation. Based on these findings, we propose that the accumulation of ROS by BA acted as a signaling factor initiating cellular responses including arrest at G2/M phase and apoptosis in U937 cells. This well supports the previous results that ROS plays a key role in the inhibition of proliferation and induction of apoptosis of various types of cancer cells by BA (Wang et al. 2017, 2019; Xu et al. 2017; Goswami et al. 2018; Shen et al. 2019).

**Figure 7.**
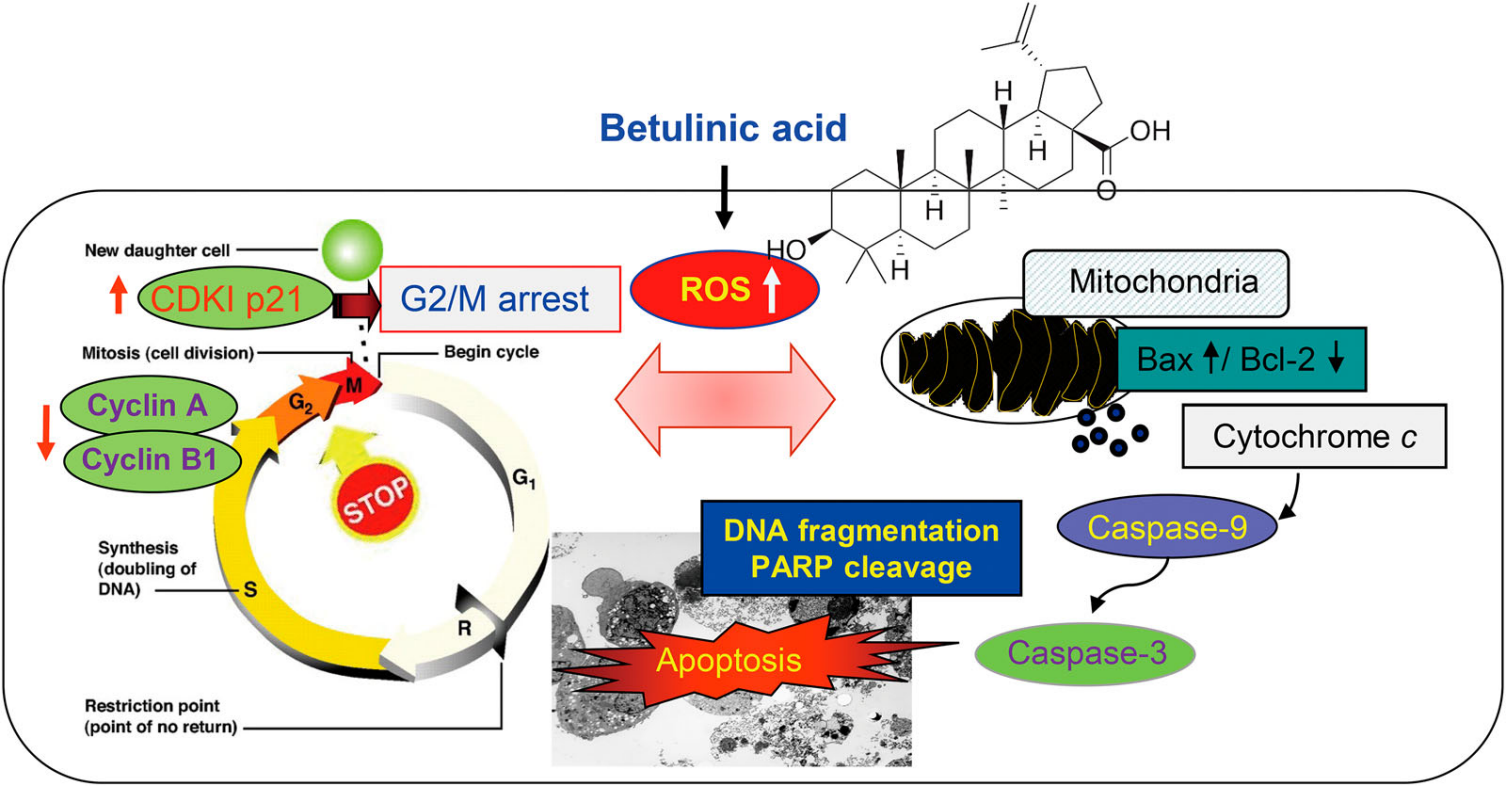
Proposed mechanism of anti-cancer potential of BA in U937 leukemia cells.

In conclusion, BA significantly inhibited survival in U937 cells compared to normal cells, and induced G2/M phase arrest and apoptosis through oxidative stress (Figure 7). However, when ROS production was blocked by the antioxidant NAC, BA-mediated anti-cancer action events were completely abolished, indicating that BA exerts anti-cancer activity against U937 leukemia cells through a ROS-dependent mechanism. Therefore, our findings suggest that BA could be developed as an effective drug for the prevention and treatment of cancer. However, further studies are needed to determine whether the production of ROS by BA is a mitochondrial damage-dependent phenomenon.
